# Ein neues Web-basiertes Verfahren zur Darstellung der Corona-Inzidenzen in Raum und Zeit

**DOI:** 10.1007/s11943-021-00288-x

**Published:** 2021-07-14

**Authors:** Ulrich Rendtel, Andreas Neudecker, Lukas Fuchs

**Affiliations:** 1 Freie Universität Berlin, Berlin, Deutschland; 2 INWT Statistics GmbH, Berlin, Deutschland; 3 Gemeinsamer Berliner Studiengang Statistik, Berlin, Deutschland

**Keywords:** Corona Inzidenz, Infektionscluster, Kartendarstellung, Choroplethen, Geokoordinaten, I10, Y91

## Abstract

Die Darstellung der räumlichen und zeitlichen Ausbreitung der Corona-Pandemie ist ein zentrales Anliegen von epidemiologischer Forschung aber auch der öffentlichen Medien. Dieses geschieht meist über Karten, die in vielen Fällen animiert sind. Die hier vorgestellte Web-Applikation benutzt ein alternatives statistisches Konzept zur Darstellung von Corona-Inzidenzen. Statt der üblichen, aber unrealistischen Annahme einer Gleichverteilung über einer Referenzfläche benutzen wir das Verfahren von Groß et al. (2020). Dieses allgemeine statistische Konzept wird hier auf die Schätzung lokaler Corona Inzidenzen angewendet. Es vermeidet die harten Sprünge an den Kreisgrenzen, die bei den üblichen Kartendarstellungen auftreten, durch eine gemeinsame Auswertung benachbarter Kreisgebiete.

Der Fokus der Darstellung liegt hier auf der Realisierung dieses Konzepts über eine frei zugängliche Web-Applikation und ihre Nutzung. Anhand von drei Beispielen wird gezeigt, dass während der zweiten Corona-Welle in Deutschland feste, lokale Cluster existieren, die sich über die Zeit auch ausbreiten und miteinander verschmelzen können.

## Abstract (en)

The representation of the spacial and temporal dispersion of the Corona pandemic is a key issue of epidemiologic research but also of public media. This issue is often realized via maps which are often animated. The web-application which is presented here uses an alternative statistical concept for the display of Corona incidences. Instead of the standard assumption of a uniform distribution over the reference area we use the approach of Gross et al. (2020). This general statistical approach is applied here for the estimation of local Corona incidences. The approach avoids the discontinuities at the borderlines of counties which appear in standard maps by a joint analysis of neighboring counties.

The focus of the presentation is the realization of this concept by a web-application and its use. By three examples we demonstrate that during the second Corona wave there exist in Germany fixed local clusters which may broaden over time and which may also merge.

## Einleitung

Die Darstellung der räumlichen und zeitlichen Ausbreitung der Corona-Pandemie ist ein zentrales Anliegen von epidemiologischer Forschung aber auch der öffentlichen Medien. Dieses geschieht meist über Karten, die in vielen Fällen animiert sind und über den Maus-Zeiger weitere Informationen zu den angezeigten Flächenstücken liefern. Zusätzlich kann man die Zeitdimension über einen Regler realisieren. Das Ziel ist die Darstellung von lokalen Corona-Hotspots und deren zeitlicher Entwicklung. Diese animierten Karten sind dann der Ausgangspunkt von Hypothesen über die Ausbreitung der Corona-Pandemie.

Die hier vorgestellte Web-Applikation benutzt ein alternatives statistisches Konzept zur Darstellung von Corona-Inzidenzen. Statt der üblichen, aber unrealistischen Annahme einer Gleichverteilung über einer Referenzfläche benutzen wir das Verfahren von Groß et al. (2020). Dieses Verfahren schätzt die unbekannten Geokoordinaten der Infektionen über einen Simulated EM-Algorithmus. Dieses allgemeine statistische Konzept wird hier auf die Schätzung lokaler Corona-Inzidenzen angewendet. Es vermeidet die harten Sprünge an den Kreisgrenzen, die bei den üblichen Kartendarstellungen auftreten, durch eine gemeinsame Auswertung benachbarter Kreisgebiete. Andere Anwendungsgebiete sind beispielsweise die Schätzung der Siedlungsgebiete ethnischer Minoritäten (Gross et al. 2017), regionale Bedarfsschätzungen zur Kinderbetreuung (Rendtel und Ruhanen 2018) oder regionale Wahlanalysen (Erfurth et al. 2021).

Der Fokus der Darstellung liegt hier auf der Realisierung dieses Konzepts über eine frei zugängliche Web-Applikation und ihre Nutzung. Anhand von drei Beispielen wird gezeigt, dass während der zweiten Corona-Welle in Deutschland feste, lokale Cluster existieren, die sich über die Zeit auch ausbreiten und miteinander verschmelzen können.

## Die Darstellung von Corona-Inzidenzen über das Robert-Koch-Institut und in den Medien

In Deutschland sammelt das Robert-Koch-Institut (RKI) die Meldungen der Gesundheitsämter zu den Neuinfektionen mit dem SARS-Cov2 Virus, die hier der Einfachheit halber als Corona-Infektionen bezeichnet werden. Die Ergebnisse werden in einem „Täglichen Lage- und Situationsbericht der RKI zu COVID-19“ veröffentlicht^1^.

In den Medien finden sich zahlreiche Darstellungen der Intensität und der räumlichen Verteilung von Corona-Neuinfektionen. Mittlerweile benutzen die meisten Darstellungen die sogenannte 7‑Tages-Inzidenz, die die Neuinfektionen auf jeweils 100 Tausend Einwohner bezieht. Die Kumulation über 7 Tage berücksichtigt die starke wöchentliche Saisonalität der Angaben der Gesundheitsämter RKI. Der Bezug auf die Einwohnerzahl erzeugt eine regionale Vergleichbarkeit zwischen Gebieten mit hohen und niedrigen Einwohnerzahlen.

Als regionale Bezugseinheit wird bei internationalen Vergleichen Deutschland als Ganzes gewählt. Für Entscheidungen zur Eindämmung der Corona-Pandemie wird meist die Ebene der Bundesländer gewählt. Die niedrigste Ebene, die für die Medien vom RKI zur Verfügung gestellt wird, ist die Ebene der Land- und Stadtkreise.

**Figure 1:**
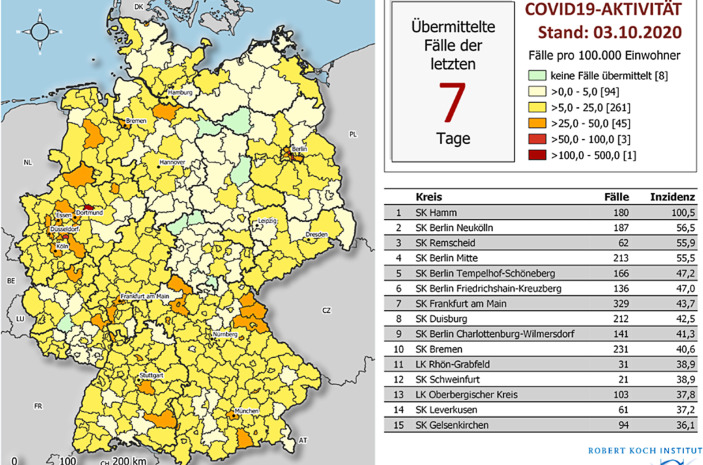

Die folgende Darstellung der 7‑Tages-Inzidenz auf Kreisebene in Abb. 1 ist typisch für fast alle Medien. Diese Darstellung des RKI zeigt die regionale Verteilung der Inzidenzzahlen an einem fixen Datum, dem 3. Oktober 2020 am Beginn der zweiten Corona-Infektionswelle in Deutschland.

In vielen Medien sind derartige Kreiskarten interaktiv. Hier liefert eine Mouse-Over-Funktion zusätzliche Informationen über den Namen des jeweils angezeigten Kreises sowie die exakte Inzidenzzahl, die bei der Farbkodierung verloren geht. Auch andere kreisbezogene Informationen, zum Bespiel die Absolutzahl der Neuinfektionen im Kreis, können zusätzlich über die Mouse-Over-Funktion angezeigt werden.

**Figure 2:**
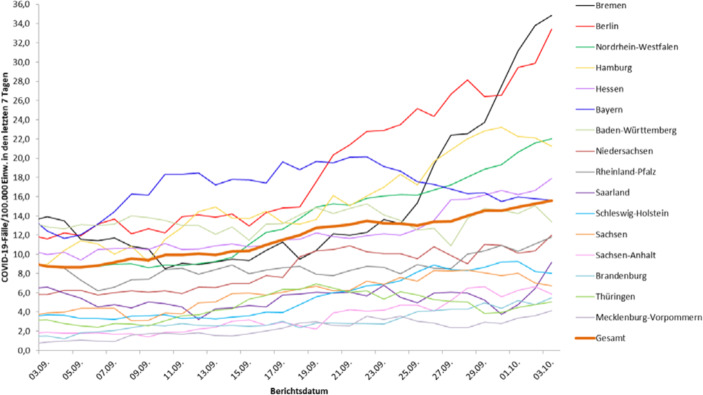

Hinsichtlich der zeitlichen Entwicklung der Inzidenz wird fast durchgängig eine Darstellung über Linienzüge gewählt. Als Darstellungsebene wird hier meist die Ebene der Bundesländer gewählt, vgl. Abb. 2. Eine Darstellung auf Kreisebene würde in einem Diagramm mit 412 Linienzügen resultieren. Derartige Diagramme sind jedoch nur schwer lesbar und damit auch schwer interpretierbar.

Eine echte Darstellung in Raum **und** Zeit ist prinzipiell über eine Kombination von Kreiskarte mit einem Zeitregler möglich. Allerdings wird diese Darstellung fast nirgendwo in den Medien und auch nicht vom RKI präsentiert. Eine Ausnahme findet man beispielsweise unter der URL https://interaktiv.tagesspiegel.de/lab/corona-analyse-in-welchen-regionen-die-zahlen-wieder-steigen/ Allerdings ist diese Darstellung wenig geeignet, die zeitliche Entwicklung von lokalen Infektionsherden zu verfolgen. Die zeitlichen Muster sind sehr instabil, so dass kaum ein visueller Eindruck von räumlichen Clustern entsteht, deren Ausbreitung sich über die Zeit ändert.

## Ein alternativer Ansatz zur Darstellung von räumlichen Infektionsclustern

Während am Anfang der Corona-Pandemie die Übertragung des SARS-Cov‑2 Virus aus Gebieten außerhalb Deutschlands im Vordergrund stand, so hat sich mit zunehmender Verbreitung des Virus im Lande auch der Ausbreitungsmodus auf Nahinfektionen verlagert. Die Ausbreitung des Corona-Virus geschieht jetzt in erster Linie über räumliche Nähe, zum Beispiel am Arbeitsplatz, in der Schule, beim Einkauf, im Restaurant, im öffentlichen Nahverkehr oder bei privaten Treffen. Dies impliziert die Existenz von lokalen Clustern mit erhöhten Inzidenzahlen. Da die getroffenen Eindämmungsmaßnahmen (Lockdown) der Corona-Pandemie darauf abzielten, Ansteckungen auch durch Einschränkungen der regionalen Beweglichkeit (Sperrung von Landkreisen, Urlaubzielen, Schließung von Hotels) zu unterbinden, ist auch mit einer gewissen zeitlichen Stabilität dieser Cluster (Hotspots) zu rechnen. Allerdings fehlte es bisher an einer geeigneten Darstellungstechnik dieser Hotspots.

Aufgrund der Verbreitungseigenschaften des Corona-Virus ist nicht mit harten Sprüngen längs der Kreisgrenzen zu rechnen. Diese Sprünge sind die Konsequenz der Annahme einer einzigen Inzidenzzahl für das gesamte Kreisgebiet. Diese Annahme teilen alle Darstellungen über Kreiskarten. Jedoch sind die vom RKI gemeldeten Infektionszahlen auch mit einer ungleichen Verteilung der Infektionsrisiken im Kreisgebiet kompatibel. Geht man von der Annahme einer stetigen Verteilung der Corona-Ansteckungsrisiken über ganz Deutschland aus, so lässt sich diese Verteilung anhand der RKI-Kreiszahlen schätzen. Hierbei werden benachbarte Kreisgebiete simultan analysiert. Das Resultat ist eine Kreis-unabhängige stetige Funktion für Deutschland, die die Anzahl der auftretenden Neuinfektionen an einem Ort beschreibt. Diese Infektionsdichte muss nur noch durch einen Schätzer für Bevölkerungsdichte normiert werden. Auf diese Weise erhält man einen lokalen Inzidenzwert. Einzelheiten dieses statistischen Verfahrens findet man in Groß et al. (2020) sowie in Erfurth et al. (2021). Das Verfahren wurde nicht direkt für die Darstellung der Corona-Inzidenzen entwickelt. Vielmehr referiert es auf eine Situation, wo für einzelne Flächenstücke nur Aggregate zur Verfügung stehen. Um eine stetige Darstellung über einen Kerndichte-Schätzer zu ermöglichen, werden die unbekannten exakten Geokoordinaten simuliert. Dies geschieht so, dass die simulierten Aggregate mit den vorgegebenen Werten übereinstimmen. Allerdings ist die resultierende Dichte nicht mehr auf den Flächenstücken gleichverteilt. Für die Realisierung kann das R‑Paket Kernelheaping^2^ verwendet werden.

**Figure 3:**
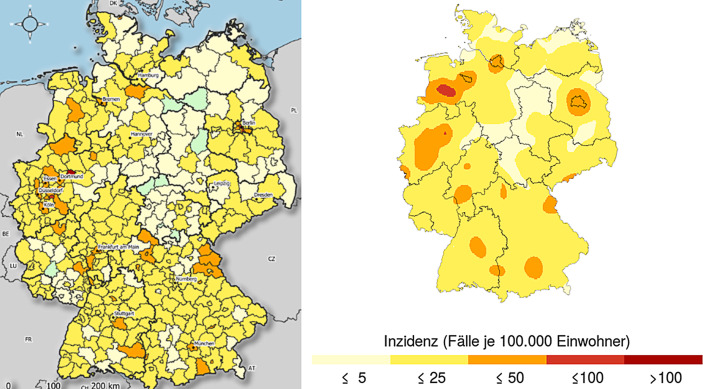

Diese lokalen Inzidenzwerte kann man wieder auf einer Karte darstellen. Beispielsweise vergleicht Abb. 3 diese alternative Darstellung mit der Kreiskarte des RKI:

Auffällig ist hierbei ein Hotspot südwestlich von Bremen (Nähe Cloppenburg), der sich über die gesamte zweite Welle als sehr stabil erweisen wird. Diese Region ist durch landwirtschaftliche Großbetriebe mit viel Tierhaltung geprägt. In einer ähnlichen Region im Münsterland war die Großschlachterei Tönnies in Rheda-Wiedenbrück im Juli 2020 durch 1413 positiv getestete Beschäftigte aufgefallen^3^. Der hier dargestellte Hotspot deutet auf ein ähnliches Risiko hin.

Weiterhin weisen alle Ballungsräume in Deutschland auf regional erhöhte Inzidenzwerte hin. Besonders deutlich ist die Ausstrahlungswirkung auf das Umland im Fall von Berlin zu sehen. In Nordrhein-Westfahlen sind die separat ausgewiesenen Stadtkreise des Ruhrgebiets in Abb. 3 zu einem einzigen Infektionsgebiet verschmolzen worden.

Ein weiterer Hotspot zeigt sich in der Oberpfalz nahe der Tschechischen Grenze. Diese Gegend war schon in der ersten Corona-Welle durch teilweise extrem hohe Inzidenzwerte (z. B. der Landkreis Tirschenreuth mit einen Inzidenzwert von 1470 am 21.04.2020) aufgefallen. Damals wurde vermutet, dass die Ursachen in Super-Spreading-Ereignissen wie dem örtlichen Starkbierfest lagen. Allerdings kann die Ansteckung im Oktober 2020 auch über die zahlreichen Pendler aus Tschechien hereingetragen worden sein. Auch dieser Hotspot wird sich durch die gesamte zweite Welle als sehr stabil erweisen.

## Die zeitliche Darstellung des Pandemieverlaufs

Um eine kontinuierliche zeitliche Darstellung der Hotspots zu ermöglichen, wurde diese Karte für ein gleitendes 7‑Tagesintervall vom Beginn der zweiten Corona-Welle (1. Oktober 2020) bis zum aktuellen Datum berechnet^4^. Diese Sequenz von zeitlich geordneten Karten wurde für eine Web-Präsentation aufbereitet, die unter der URL https://www.inwt-statistics.de/blog-artikel-lesen/COVID-19_Karte_der_lokalen_7-Tage-Inzidenz_im_Zeitverlauf.html frei zugänglich ist.

Die Darstellung kann über Regler wie ein Video gesteuert werden. Buttons erlauben es, in der Zeit um einen einzelnen Tag vor- und zurückzugehen, um Hotspots besser verfolgen zu können.

**Figure 4:**
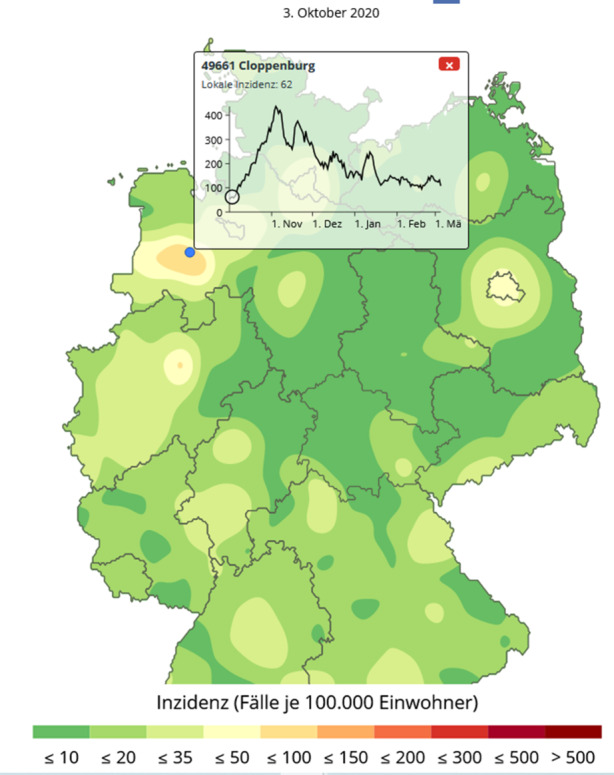

Die Karte verfügt zunächst über keine Ortsangaben, weil ihre Einblendung die Wahrnehmung der örtlichen Cluster stören wurde. Aus diesem Grund wurde eine Mouse-Over-Funktion benutzt, mit der Orte und ihre zeitlichen Inzidenzprofile dargestellt werden können. Man kann den Mauszeiger über die Karte führen und erhält die Ortsnamen mit Postleitzahl. Durch Klick, zum Beispiel im Zentrum eines dargestellten Hotspots, erhält man dann eine Information über den zeitlichen Verlauf der Inzidenzzahlen an diesem Ort. Abb. 4 zeigt den steil ansteigenden Infektionsverlauf für die Stadt Cloppenburg, der nur langsam abfällt und noch im März 2021 deutlich über der Marke von 100 liegt.

**Figure 5:**
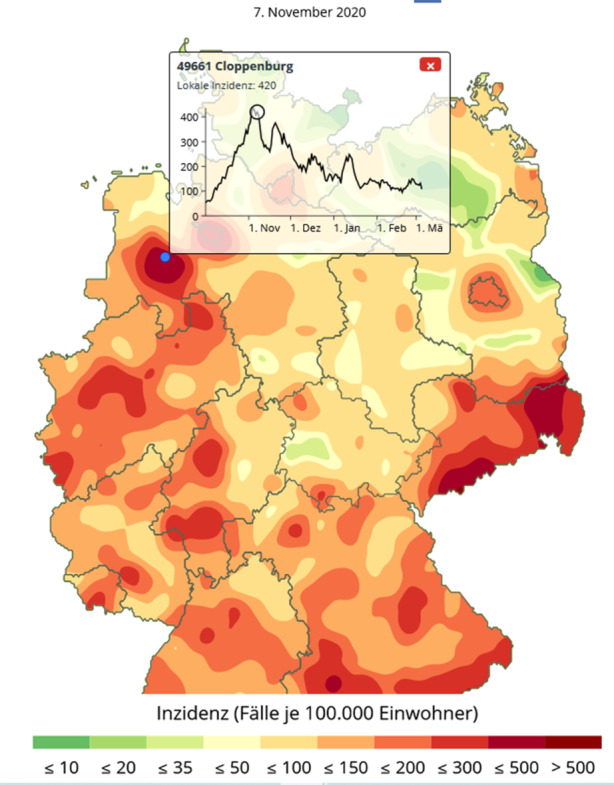

Durch Anklicken eines späteren Zeitpunkts, zum Beispiel des Zeitpunkts mit der höchsten Inzidenz, wechselt die Hintergrundkarte auf die Situation an dem angegebenen Zeitpunkt, vgl. Abb. 5. Man sieht, dass die Stadt Cloppenburg auch noch nach einem Monat in einem der „heißesten“ Inzidenzgebiete Deutschlands liegt. Allerdings hat sich das Niveau der Neuerkrankungen von einem Inzidenzwert 62 auf 420 fast versiebenfacht.

**Figure 6:**
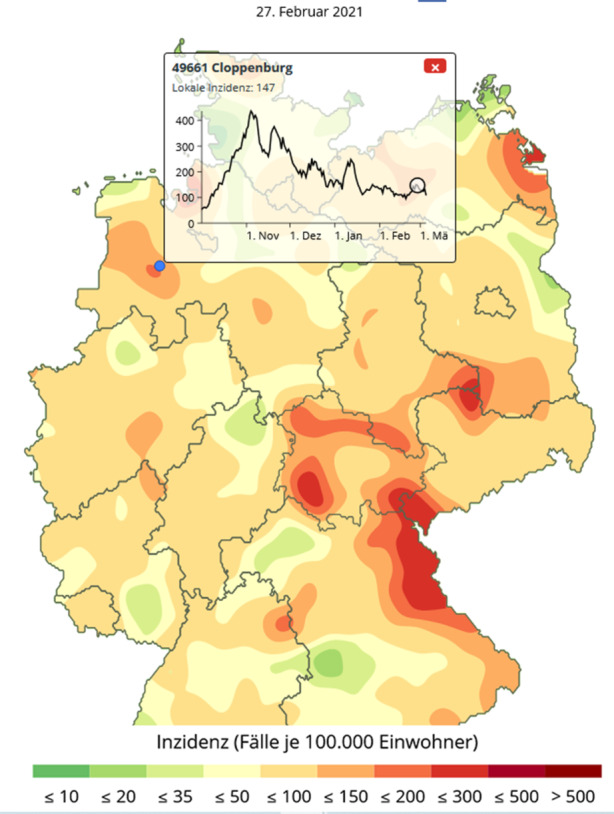

Selbst in der auslaufenden zweiten Infektionswelle bildet die Gegend um Cloppenburg noch einen lokalen Hotspot, vgl. Abb. 6. Diese zeitliche Stabilität der regionalen Hotspots ist bemerkenswert und wird über die bisherigen Darstellungsformen nicht vermittelt.

**Figure 7:**
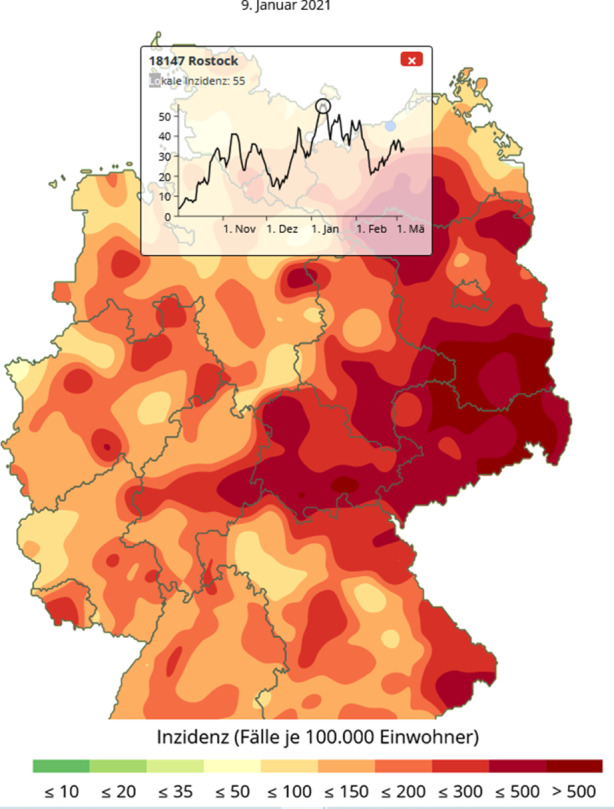

Ebenso gut kann man mit dieser Darstellungstechnik auch „Cold-Spots“ (also Gebiete mit geringer Inzidenz) identifizieren. Hier spielt die Stadt Rostock eine für Deutschland herausragende Rolle. Wie die Abb. 7 zeigt, sind die lokalen Inzidenzwerte während der gesamten zweiten Infektionswelle bis auf geringe Abweichungen unter dem Wert 50 geblieben. Auf dem Höhepunkt der Infektion ist Rostock von Infektionsgebieten mit deutlich höheren Inzidenzen umgeben.

**Figure 8:**
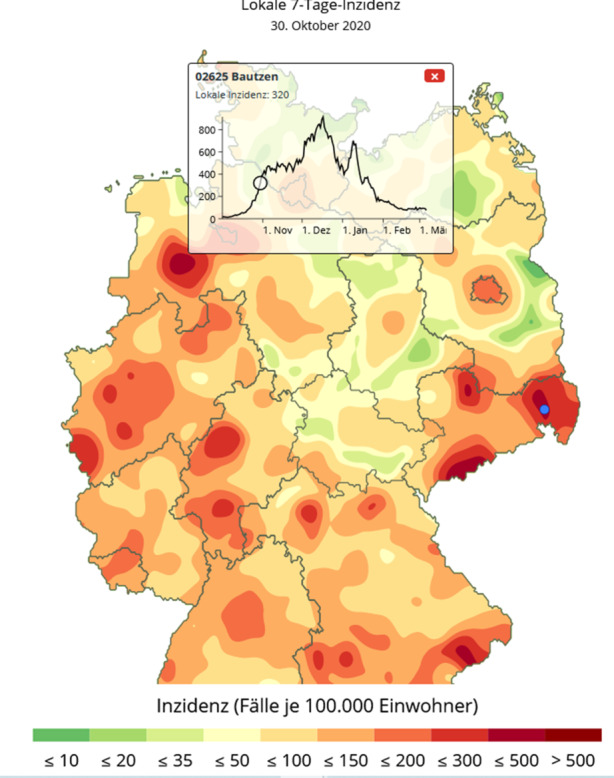

**Figure 9:**
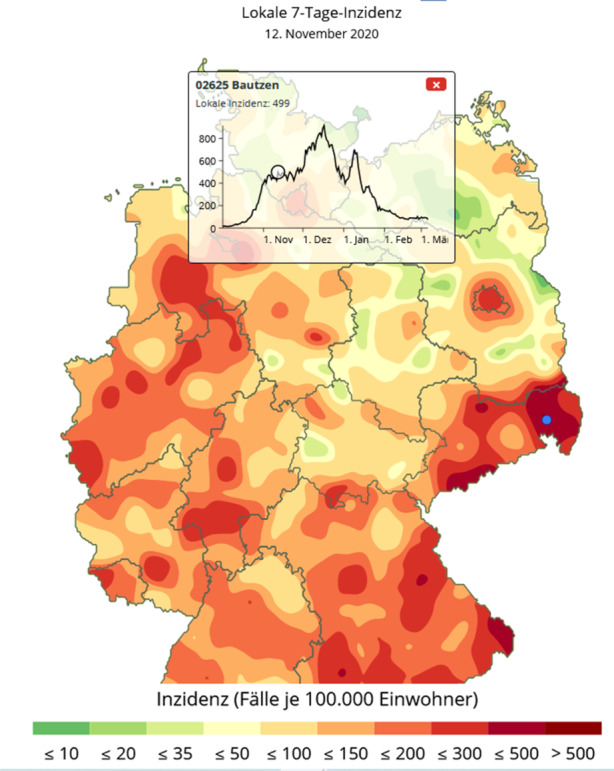

**Figure 10:**
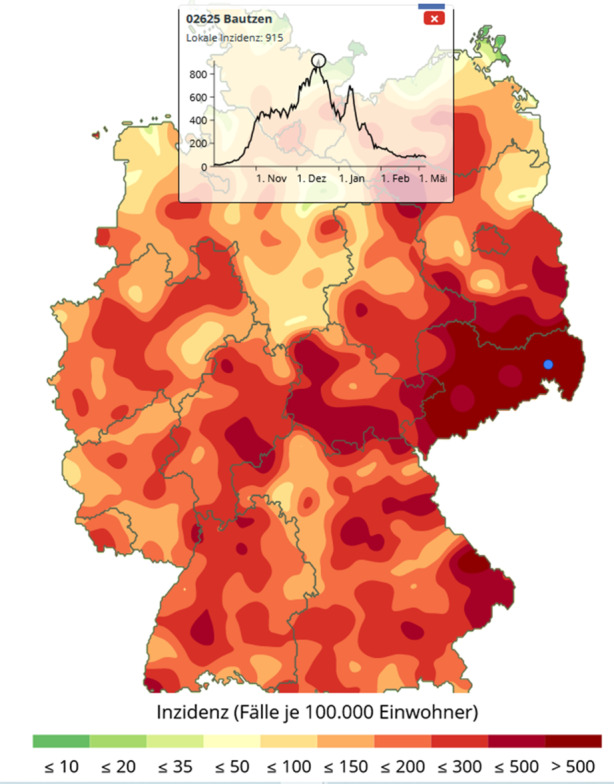

Daneben zeigen Infektionsgebiete auch ein dynamisches Verhalten: So können separate Hotspots sich in kurzer Zeit vergrößern und auch miteinander verschmelzen. Beispielsweise zeigten sich am 30. Oktober drei separate Infektionskerne um die Stadt Bautzen, am Rand des Erzgebirges um Annaberg und nördlich davon in der Stadt Riesa an der Elbe, vgl. Abb. 8. Knapp 2 Wochen später haben sich diese Infektionsherde vereinigt und bilden eine gemeinsames Infektionsgebiet, das nur noch die Großstädte Dresden und Chemnitz ausspart, vgl. Abb. 9. Einen Monat später ist fast der gesamte Freistaat Sachsen ein Corona-Hotspot mit einer Inzidenzzahl von 432, vgl. Abb. 10.

## Die Nutzung der Darstellung von Infektionsclustern

Mit der hier beschriebenen Web-Applikation lassen sich für Deutschland große Unterschiede bezüglich der Corona-Inzidenzzahlen zeigen. Diese Hotspots können räumlich stabil sein oder sich auch dynamisch ausbreiten.

Die Lage und die zeitliche Entwicklung der Hotspots geben Anhaltspunkte für mögliche Gründe der Ausbreitung des Corona-Virus. Im Fall von Cloppenburg könnte man nach ähnlichen Ursachen wie bei der Großschlachterei Tönnies^5^ forschen. Der Cold-Spot Rostock ist bereits durch seine frühzeitige und konsequente Nutzung von Corona-Tests bekannt geworden^6^. Der Fall Sachsen fällt zunächst durch den Start der Infektionen in unmittelbarer Nähe zur Tschechischen Grenze auf. Da Tschechien im Oktober/November im Landesdurchschnitt Inzidenzen bis zu 800 hatte, könnte hier eine Infektion über Berufspendler stattgefunden haben. Allerdings gibt es auch Berichte über einen deutschen Biertourismus nach Tschechien^7^, das in seinen Lockdown-Regelungen nicht so streng war wie Deutschland. Schließlich gab es auch Vermutungen über eine Assoziation von politischen Präferenzen, die mit einer Ablehnung von Corona-Hygiene-Vorschriften einhergehen und zu einem erhöhten Corona-Ansteckungs-Risiko führen^8^.

Allerdings beschränkt die regionale Auflösung der verwendeten Aggregatszahlen die Interpretation der gefundenen Cluster. Hierbei ist die Unterteilung Deutschlands in 412 Landkreise und Stadtbezirke sicher eine gute Basis für Interpretationen auf nationaler Ebene. Allerdings gestatten beispielsweise die 12 Aggregate der Berliner Stadtbezirke keine separaten Analysen für das Berliner Stadtgebiet^9^^,^^10^. Hier wäre der Zugang zu Aggregaten auf kleineren regionalen Einheiten wünschenswert. Solche Zahlen werden jedoch vom RKI nicht bereitgestellt.

Einige Cluster liegen direkt an der Grenze zu Österreich beziehungsweise Tschechien. Hier ist es wünschenswert die Ausdehnung der Infektionscluster über die nationalen Grenzen darzustellen und gegebenenfalls die Dynamik an den nationalen Grenzen zu überprüfen. Dies plädiert für eine eher europäische Sichtweise. Klassische Kartendarstellungen^11^^,^^12^ zeigen, dass sich die Corona-Pandemie in Europa zeitlich und räumlich sehr ungleich ausgebreitet hat. Allerdings würde die Realisierung einer Europakarte mit der hier benutzten statistischen Methodik auf Kapazitätsgrenzen bei der Auslastung der Rechner^13^ stoßen. Für Grenzregionen, beispielsweise zu Tschechien oder die Alpenregion, erscheint ein solcher Ansatz jedoch realisierbar. Allerdings sind die Inzidenzzahlen von den örtlichen Testungsstrategien abhängig. Diese können in den einzelnen Ländern stark voneinander abweichen und so in unterschiedlichen Inzidenzzahlen resultieren. Dies erschwert einen Vergleich über die Landesgrenzen.

## Resüme

Die hier vorgestellte Web-Applikation ermöglicht für Deutschland die Identifikation von räumlichen Corona-Infektionsclustern und deren zeitlicher Entwicklung. Sie behebt damit einen Mangel der üblichen Darstellungen über Kreiskarten. Die Darstellung basiert zwar auf denselben Kreisaggregaten des RKI, benutzt aber nicht die unrealistische Annahme einer gleichmäßigen Verteilung der Corona-Infektionen über das gesamte Kreisgebiet. Die Ergebnisse zeigen regional stabile interpretierbare Corona-Infektionscluster.

Die Web-Applikation ist frei verfügbar und wird ständig aktualisiert.

## Footnotes

1 https://www.rki.de/DE/Content/InfAZ/N/Neuartiges_Coronavirus/Situationsberichte/Gesamt.html.

2 https://cran.r-project.org/web/packages/Kernelheaping/index.html.

3 https://www.wa.de/nordrhein-westfalen/coronavirus-toennies-rheda-nrw-schlachthof-mitarbeiterin-rtl-arbeitsbedingungen-lockdown-zr-13755098.html (Download 23.03.2021).

4 Die Darstellung wird täglich automatisch aktualisiert. Die Copyright Lizenz ist CC BY-NC-SA 4.0 (https://creativecommons.org/licenses/by-nc-sa/4.0/legalcode Download 25.05.2021).

5 https://de.wikipedia.org/wiki/T%C3%B6nnies_Holding#Ausbruch_im_Stammwerk_Rheda-Wiedenbr%C3%BCck (Download 21.03.2021).

6 https://www.deutschlandfunkkultur.de/oberbuergermeister-claus-ruhe-madsen-erfolgreiches.1008.de.html?dram:article_id=489976 (Download 21.03.2021).

7 https://www.mdr.de/nachrichten/welt/osteuropa/politik/tschechien-corona-who-babis-100.html (Download 21.03.2021).

8 https://www.rnd.de/politik/viel-corona-viel-afd-alles-zufall-OQVV5N5SH5DIHAOZQPFU6MZDPA.html (Download 21.03.2021).

9 https://www.berlin.de/corona/lagebericht/ (Download 21.322.021).

10 https://www.berlin.de/sen/gesundheit/service/gesundheitsberichterstattung/veroeffentlichungen/kurz-informiert/#Corona20 (Download 21.03.2021).

11 https://experience.arcgis.com/experience/3a056fc8839d47969ef59949e9984a71 (Download 23.03.2021).

12 https://jeodpp.jrc.ec.europa.eu/services/shared/Videos/cvEuropeRegions7daysNewCases.mp4 (Download 23.03.2021).

13 Immerhin wird bei diesem Verfahren die Geokoordinate eines jeden Einwohners des analysierten Gebiets bei jedem Iterationsschritt des Algorithmus simuliert. Für Deutschland benötigte die Erstellung einer einzigen Inzidenzkarte bei einer Rechnerleistung von 30 GB RAM und einem CPU-Kern ungefähr 3:20 h für 7 Tage, also eine knappe halbe Stunde pro Tag. Es werden wegen möglicherweise fehlenden Daten immer die letzten 7 Tage berechnet.
